# Analytic Correlation Filtration: A New Tool to Reduce Analytical Complexity of Metabolomic Datasets

**DOI:** 10.3390/metabo9110250

**Published:** 2019-10-24

**Authors:** Stephanie Monnerie, Melanie Petera, Bernard Lyan, Pierrette Gaudreau, Blandine Comte, Estelle Pujos-Guillot

**Affiliations:** 1 Université Clermont Auvergne, INRA, UNH, Mapping, F-63000 Clermont Ferrand, France; blandine.comte@inra.fr; 2 Université Clermont Auvergne, INRA, UNH, Plateforme d’Exploration du Métabolisme, MetaboHUB Clermont, F-63000 Clermont-Ferrand, France; melanie.petera@inra.fr (M.P.); bernard.lyan@inra.fr (B.L.); 3 Centre de Recherche du Centre Hospitalier de l’Université de Montréal, Montréal, QC H2X 3E4, Canada; pierrette.gaudreau@umontreal.ca; 4 Département de Médecine, Université de Montréal, Montréal, QC H3T 1J4, Canada

**Keywords:** metabolomics, data filtration, high-resolution mass spectrometry

## Abstract

Metabolomics generates massive and complex data. Redundant different analytical species and the high degree of correlation in datasets is a constraint for the use of data mining/statistical methods and interpretation. In this context, we developed a new tool to detect analytical correlation into datasets without confounding them with biological correlations. Based on several parameters, such as a similarity measure, retention time, and mass information from known isotopes, adducts, or fragments, the algorithm principle is used to group features coming from the same analyte, and to propose one single representative per group. To illustrate the functionalities and added-value of this tool, it was applied to published datasets and compared to one of the most commonly used free packages proposing a grouping method for metabolomics data: ‘CAMERA’. This tool was developed to be included in Galaxy and is available in Workflow4Metabolomics.

## 1. Introduction

Metabolomics is described as an approach allowing the description of small molecules/metabolites present in a biological system by identifying and possibly quantifying them [1]. By the global study of low molecular weight compounds, it allows determining metabolic phenotypes that may vary between individuals depending on several factors: genetics, environment exposure, and life habits. As it gives an integrated vision of the health status, metabolomics has been shown in recent years as a powerful tool to better understand the biological mechanisms involved in the pathophysiological processes and to identify disease biomarkers [2]. However, this approach generates massive and complex data that need adequate analyses to extract the biologically meaningful information.

In metabolomics, mass spectrometry (MS) is one of the most common analytical platforms. Upstream to the ionization and detection steps, metabolites from complex biological samples are separated most frequently using liquid chromatography (LC) or gas chromatography (GC) [3,4]. During the analysis, metabolites are ionised in the mass spectrometer source to produce several ions/analytical features (parent ion, isotopes, adducts, and fragments) that are part of the original molecule. Raw data obtained from metabolic profiles are processed to yield a data matrix containing retention times (time between sample injection and appearance of the maximum ion signal), masses, and peak intensities. This step results in thousands of features present in the final dataset with a high degree of correlation [5]. In addition to this analytical redundancy, biological correlation issued from modulated metabolites coming from the same pathways does also exist. This high degree of correlation in datasets is a constraint for the use of various data mining and statistical methods. For example, analytical redundancy highly affects multiple testing correction. Indeed, having non-independent variables (coming from the same metabolite) leads to an over-correction of data that can hide potentially relevant information. Moreover, for biological interpretation, experts are mainly focusing on metabolites rather than on the different analytical features. For all these reasons, considering metabolite as a unique entity instead of the individual ions, as a variable, is more relevant.

In order to handle this data complexity and, in particular, identify subgroups of related features, tools have been developed, mostly for annotation purposes [6]. In fact, the use of correlations between ions contributes to determining chemical identities and isolating species part of the same metabolite from those of other coeluting compounds. Generally, the available packages consist of a two-step process: first, a grouping of all features derived from the same analyte, and then, an annotation of the ion species. The first category of tools is based on a grouping approach consisting of using chromatographic peak-shape similarity of coeluting features in raw data. Among these tools, CAMERA is a Bioconductor R package that is widely used in the field of mass spectrometry metabolomics [7], designed to post-process XCMS [8] feature lists and to collect all features related to a compound. In an iterative process, it first selects the most intense feature not yet assigned to a compound spectrum and determines an associated retention time (RT) window. All features within this range are then included in a new compound spectrum. Next, the algorithm excludes unfitting features using the chromatographic peak shape similarity of the extracted ion chromatogram (EIC) of each feature, as well as a Pearson correlation between intensities inside the chromatographic peak boundaries for all pairs of features within the compound spectrum. Finally, in the last step, it allows annotation of adducts, common neutral losses using a combination of lists of observable ions. Alternatively, some computational tools involve intensity correlation analysis across multiple samples as the basis of feature grouping. AStream [9], a tool designed for LC/MS annotation, is based on a grouping method using intensity correlations, retention time, and adduct, isotope, and fragment identification. This tool, available as an R package, uses the intensity correlations across samples for all the features present in the data instead of analysing the individual chromatogram correlations. Therefore, it does not require the raw LC/MS data but only features intensities. More recently, cliqueMS [10] proposed an integrated approach building a feature similarity network from coeluting profiles. xMSannotator [11] also incorporates multi-criterion scoring (both analytical and biological correlations, matches in databases, etc.) for improving the annotation of high-resolution metabolomics data.

Even though a grouping step does exist in these packages, most of the time all the generated information (correlation coefficient, retention time, mass difference between features) are not used together to form the groups. Moreover, users do not have the possibility of accessing the grouping information nor obtaining representative features among groups, which is essential for data reduction. Finally, most of those tools are not giving the possibility to build a workflow with other processing tools, as they require either a specific input format or specific approach for measuring similarities.

In the context of contemporary e-Science, it has been recognised that data have to be Findable, Accessible, Interoperable, and Reusable in the long-term [12]. Workflow4Metabolomics (W4M, http://workflow4metabolomics.org; [13,14]) has been developed within this objective, for comprehensive metabolomics data pre-processing, statistical analysis, and interpretation. It is a fully open-source virtual research environment (VRE; [15]) built upon the Galaxy environment [16] for bioinformatics developers and metabolomics users and allows user-friendly functionalities for workflow management.

In this context, we proposed a new stand-alone tool dedicated to data reduction based on the removal of analytical redundancies of MS-based metabolomics datasets. As a key element within the metabolomics data analysis workflow, as well as to ensure reproducible computational analyses, we made it available via Galaxy and provided it for W4M, with generic input files and different output files for visualisation and further data analysis steps within workflows.

## 2. Materials and Methods

Our aim was to detect analytical correlations into MS-based metabolomics datasets (tabular files) without confounding them with biological ones that may exist within samples. To achieve our goal, we developed a Perl tool supported by metabolomics experts to translate and understand the chemical complexity of datasets as well as possible.

The algorithm principle is to group features coming from the same metabolite and to suggest one single representative per group. In optimal settings, the grouping criteria include a similarity measure, retention time, and mass information from a reference list containing isotopes, adducts, and fragments. Thresholds for all these criteria can be fixed, and the representative feature can be determined following four methods according to the user’s needs and the analytical technology used, either LC- or GC-MS. As the output, the module returns the input file with new columns in relation to resulting groups (representative feature choice, grouping information, and annotation of features), as well as a .sif file allowing correlation network visualisation of the dataset of interest. The present tool “Analytic correlation filtration” (ACorF) is available via the web interface Galaxy as a single module and can be chained with other W4M modules.

As CAMERA is also available in W4M; the present tool was compared to this package by using a published dataset, demonstrating its utility and various possibilities of use.

### 2.1. Algorithm Description

**Figure 1:**
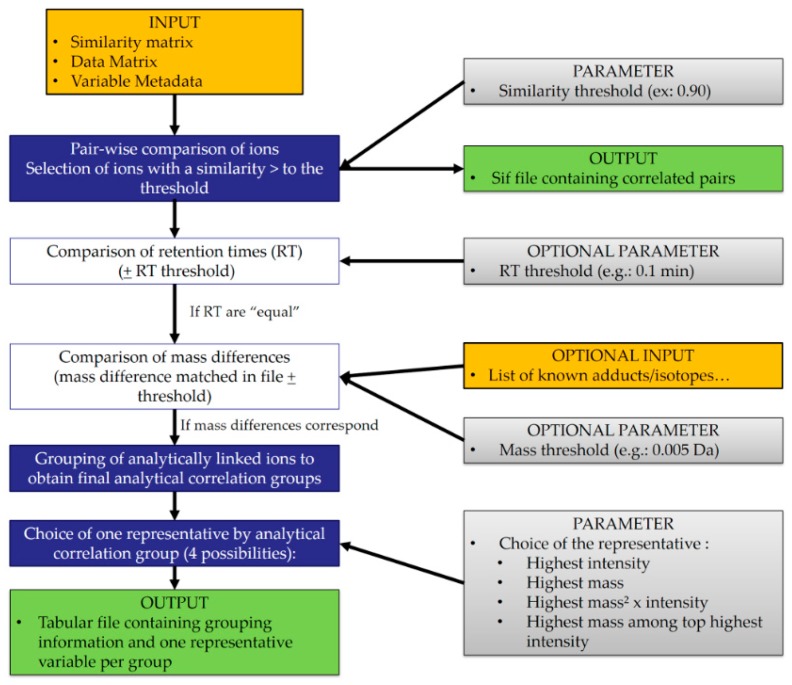
Flowchart representing the major steps of the algorithm (input and output files, parameters, and calculation steps). The key mandatory steps are filled in blue, while optional ones are in white.

Major steps of the algorithm are presented in Figure 1. Source code is freely available for download under CeCILL 2.1 license at https://services.pfem.clermont.inra.fr/gitlab/grandpa/tool-acf and implement in Perl.

#### 2.1.1. Input Files

The ACorF tool takes 3 files related to collected data as input, in tabular format (see Supplemental Figure S1). The first file, referred to as data matrix, consists in a table containing intensities of each variable (each ion detected on the mass spectrum) per sample; the second file, referred to as variable metadata, consists in descriptive additional metadata of variables (e.g., m/z, retention time). The tool also takes, as input, a third file, the similarity matrix: a table representing pair-wise similarity within the dataset, in CSV or tabular format. This table generation is not included in the tool to allow more flexibility: there is a large variety of similarity measures (Pearson/Spearman correlation, Clustering, Partial correlation, et al.), whose relevance can vary depending on the filtering goal. The similarity matrix can be obtained either using W4M (e.g., Metabolites Correlation Analysis, Between Table Correlation, et al.) or any external tool.

The last file, containing a list of known adducts, fragments, and isotopes, and their associated masses, is needed when choosing the mass comparison option.

#### 2.1.2. Processing

The first step of the algorithm is performing a pair-wise comparison of the different variables. The similarity matrix is read, and only pairs having a similarity coefficient higher than the chosen threshold are selected.

The next two steps are optional but highly recommended to increase analytical relevance. In a pair-wise process once again, the retention times of variables within the selected pairs are compared. If the ions have an identical RT (more or less a delta fixed by the user), their mass difference can be taken into account. Indeed, the user can specify the use of a list of known isotope, adduct, and fragment mass differences. In case the user does not provide a personal uploaded list, a default one is available within ACorF. The mass difference between two variables is compared to this list with a tolerance defined by the user, to confirm the chemical link between them. If a match is found, the two ions are considered as coming from the same metabolite and will be put in the same group. Those steps are repeated for each selected pair to obtain analytical correlation groups.

The last step consists of choosing a representative variable for each group. The user can choose among four options to allow the best choice of the quantifier depending on its technology and method (ensuring good signal to noise ratio and specificity).

(1) Retaining the ion with the highest intensity
(2) Retaining the ion with the highest mass
(3) Retaining the ion with the highest ‘mass² × average intensity’
(4) Retaining the highest mass among the top highest average intensities of the group. For this last option, the user determines the number of ions considered in the top list (top 5, top 3, top 10, etc.).

#### 2.1.3. Output Files

The correlated pairs are used to create the first output, a *.sif file containing pair-wise correlation rate. This file allows correlation network visualisation using tools such as Cytoscape [17].

Then, ACorF returns a second output file consisting in the variable metadata file (in tabular format) with additional result columns. This new file includes (i) an ‘ACorF_groups’ column that contains the group name; (ii) a ‘isotopes_adducts_fragments’ column that proposes annotations based on the list of known isotope, adducts, and fragments (relatively for each ions contained in the same group); (iii) a column entitled ‘ACorF_filter’ that indicates if the variables have to be conserved or deleted for a filtration step; (iv) a ‘representative’ column that contains the name of the variables selected as representatives of their correlation groups (the name of this column will indicate the chosen representative option); and finally (v) an ‘annotation_relative_to_representative’ column that proposes annotation of the ion comparatively to the representative ion selected for the concerned group. If no analytical correlation is found for a given ion, it is assigned to an individual group, and remaining cells are filled with ‘-’.

### 2.2. Examples of Use

To illustrate the ACorF functionalities and the results obtainable on typical experimental data, datasets publicly available on W4M were used as examples.

The first dataset, named ‘Sacurine’ (W4M00002_Sacurine-comprehensive), was obtained from LC-HRMS analyses (negative ionisation mode) of human urine samples in Guitton et al. [13] (DOI:10.15454/1.481114233733302E12). The present test was performed on a subset of the initial 7456 ion dataset after noise elimination. This subset contains a total of 184 samples and 3120 ions after various steps of pre-processing using XCMS and noise filtration. The ACorF tool was applied using parameters as close as possible to the ones of CAMERA to allow a better comparison: (i) a Pearson correlation as similarity measurement with a threshold of 0.75 (as it is used as default setting in CAMERA); (ii) a RT threshold of 0.1 min; (iii) the default list of known adducts and isotopes, with (iv) a mass threshold of 0.002 Da, and (v) the representative ion selected as the one with the highest intensity.

The second dataset, named ‘Algae’ (W4M00004_GCMS-Algae), was obtained from GC-MS analyses of algae samples (DOI:10.15454/1.4811272313071519E12) in Guitton et al. [13]. This dataset contains a total of 12 samples and 2908 ions after various steps of pre-processing using XCMS. The ACorF tool was applied using GC-MS recommendations: (i) a Pearson correlation as a similarity measurement with a threshold of 0.90; (ii) a RT threshold of 0.1 min; (iii) a list of known adducts and isotopes, with (iv) a mass threshold of 0.2 Da due to low resolution of the quadrupole mass spectrometer, and (v) the representative ion selected as the one with the mass among those with highest intensity.

## 3. Results and Discussion

### 3.1. Functionalities

We chose to compare the present tool to one of the most commonly used free packages proposing a grouping method and available as a W4M module: ‘CAMERA.annotate’.

Before comparing the results, the functionalities of both tools were listed in Table 1. The first important difference concerns the input format. The CAMERA R package requires the use of XCMS to pre-process raw data. Therefore, the CAMERA.annotate module takes an .RData as the input file resulting from the xcmsfill.peaks function. The advantage of the ACorF tool is to propose more universal inputs with three files that can easily be generated from any type of metabolomics data, whatever the software used to obtain the data. Another difference concerns the algorithm itself and in particular, the grouping step. To group ions, CAMERA defines an RT window for each peak, based on the highest intensity ion chromatogram, using 2 different parameters ([sigma] and [perfwhm]) related to the chromatographic peak characteristics. Even if the user can optimise those parameters, CAMERA does not give the possibility to have an overview of their impact on each defined peak. The present tool is based on a different algorithm that proposes to the user to determine an RT window. In addition, in CAMERA, a list of mass differences for known adduct/isotopes is not used for the pc-group formation but only for annotation purposes. In ACorF, the user can choose a default list or his own list to group ions together with similarity and RT criteria to validate the fact that the redundancy observed most likely has an analytical origin.

Finally, the major attractive feature of the present tool compared to CAMERA is its selection of a representative ion for each formed group, based on 4 alternative methods. This representative ion proposition allows dataset filtration by removing analytic correlations.

### 3.2. Example of Use: The Sacurine Dataset

To illustrate the ACorF functionalities and the results obtainable on typical experimental data, the ACorF tool was applied to the Sacurine dataset, using parameters as close as possible to the ones of CAMERA to allow a better comparison.

**Figure 2:**
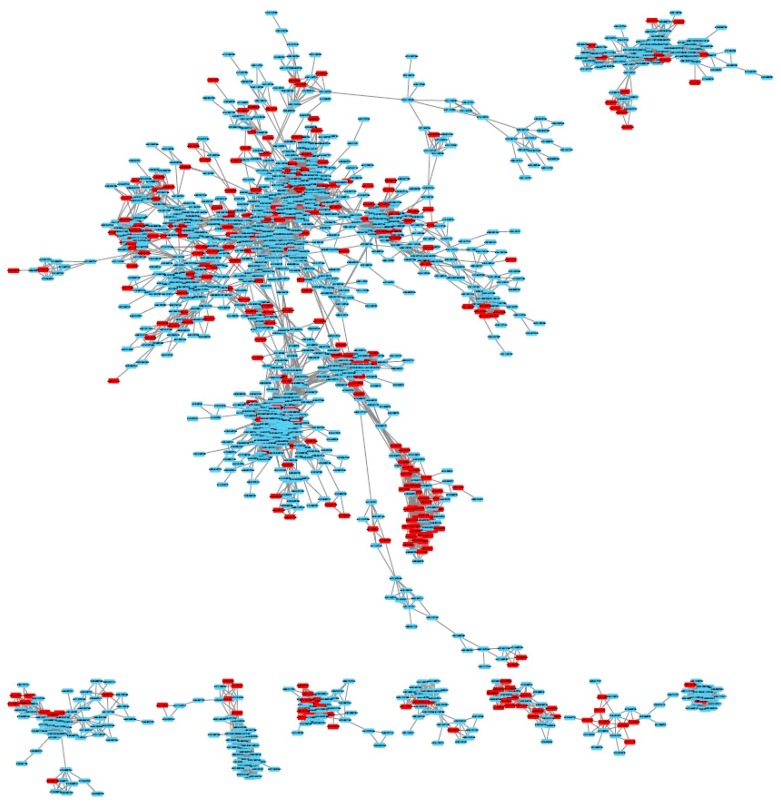
Correlation network of ions that have correlation coefficients above 0.75 in the Sacurine dataset. This network was obtained with Cytoscape using the *.sif output file of the ACorF tool. It represents groups containing more than 10 ions. Red features are identified as being redundant and tagged as deleted by the ACorF tool.

Within the 3120 ions, ACorF allowed the creation of 2697 groups, meaning that 14% of ions are proposed to be filtered from the dataset because of analytical redundancies. Using the generated *.sif file, a quick network visualisation was performed with Cytoscape (Figure 2). It represents the existing correlations >0.75 between features for groups containing more than 10 ions. An overview of all the correlations >0.75 existing in the analysed subset is available in Supplemental Figure S2.

### 3.3. Result Comparisons

To illustrate the performance of the developed tool, we compared its results obtained using the Sacurine dataset to those obtained using CAMERA.annotate in the published workflow.

**Figure 3:**
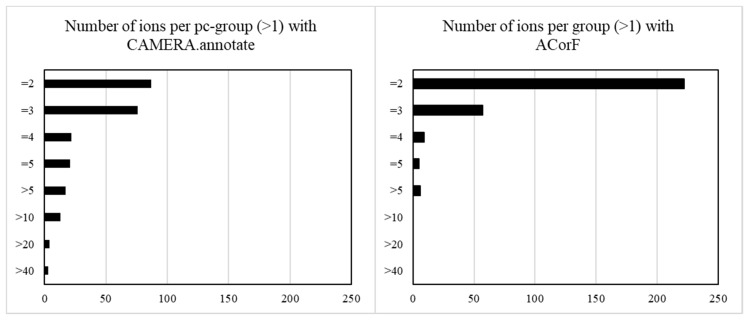
Bar diagram presenting the number of groups (x-axis) by the group size (y-axis: number of ions per group).

While CAMERA proposed 2238 pc-groups, ACorF proposed 2697 groups. With CAMERA, 89% of the pc-groups contained only one ion (1995) versus 77% for ACorF (2397). The numbers of ions in the other groups for both tools are presented in Figure 3. These histograms showed that the groups formed using the ACorF tool are smaller than the ones obtained from CAMERA. While CAMERA generated more than 20 groups of more than 10 ions, the proposed tool subdivided them into smaller ones corresponding to individual annotated metabolites.

**Figure 4:**
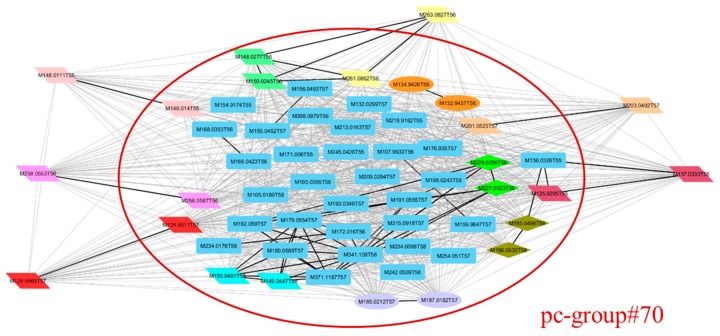
Correlation network of the largest pc-group formed by CAMERA (pc-group#70) shown in the red circle. Ions in blue are those put in individual groups by the ACorF tool. Other coloured ions are those put into groups of ≥2 ions by ACorF. Squared nodes are those that are not grouped by ACorF; ellipse nodes are grouped by considering correlation coefficient only, diamond nodes are grouped considering correlation coefficient + retention time; and parallelogram nodes are grouped considering correlation coefficient + retention time + mass differences.

To explore these results, we focused on the largest group formed by CAMERA, the pc-group#70, containing 50 ions, all eluting at the beginning of the chromatogram (small values of RT). With the ACorF tool, this pc-group is divided into 44 groups using all the parameters. Among those groups, 32 are single-ion ones that could not be linked to other ions as being part of the same metabolite. The other 12 resulting groups of 2 or more ions are presented in Figure 4: six of those groups are fully included into the initial pc-group#70, and the 6 others include additional ions from other pc-groups, raising the interest of the present approach.

To go deeper into interpretation, the impact of the 3 major steps of the ACorF algorithm was evaluated regarding this group subdivision. When only using the correlation as grouping criteria, 24 groups are formed, whereas 39 are created when adding the RT criteria and 5 more are obtained using the mass difference parameter. In this early-eluted compound area, many metabolites are detected within a very narrow RT, increasing the interest of taking this RT factor into account in the grouping step. This point was also highlighted on identified compounds from plasma samples [18] (see Supplemental Figure S3).

To illustrate the added value of the mass difference parameter, we focused on the pc-group #93 obtained from CAMERA that contains 15 of more retained ions, eluting at around 5 min.

**Figure 5:**
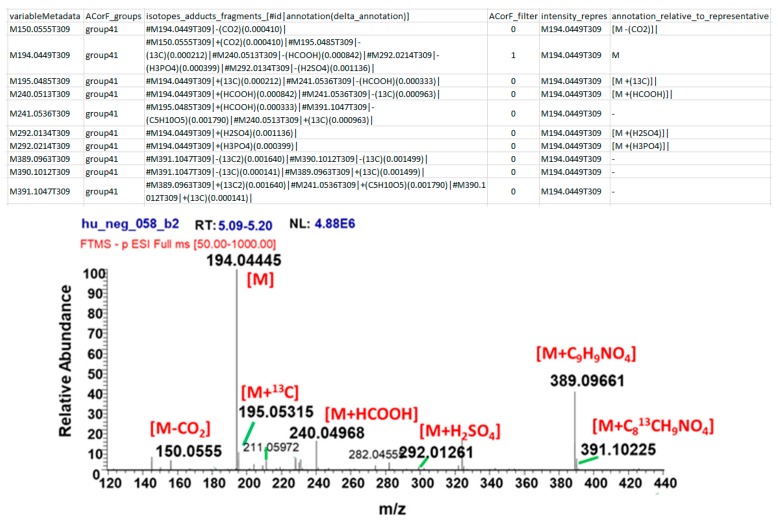
Example of annotation of mass difference between features using ACorF (mass threshold 0.002 Da) and the comparison of an expert annotated raw spectrum.

ACorF divided the pc-group#93 into 8 groups using all parameters. In particular, the sub-group#41 contains 10 annotated ions: 8 within the pc-group#93 and 2 more that are included in the pc-group#2729 (M292.0134T309 and M292.0214T309). An example of the annotation of mass defect between features using ACorF (mass threshold 0.002 Da) is presented for sub-group#41 in Figure 5 and compared to an expert annotated raw spectrum, illustrating the validity of the mass defect grouping. Moreover, on this particular example, ACorF allowed for the annotation of sulfate and phosphate moieties that are not identified by CAMERA.

On a global point of view concerning the Sacurine dataset annotation, ACorF provided in-source annotation from the ions of the dataset for 100% of the grouped variables, as it is one parameter of the grouping process, whereas CAMERA proposed annotation using hypothetic observable ions for 70% of its grouped features.

### 3.4. Use and Configuration

ACorF can be used to process either LC- or GC-MS data; recommendations for different parameter settings for a successful filtration are the following:

For UPLC/HRMS data, default parameters can be the following: (i) if a Pearson correlation is used, the default threshold can be set at 0.90; (ii) a delta RT of 0.1 min or adjusted depending on chromatographic systems; (iii) the use of the list of known adduct/isotope mass differences with a mass delta of 0.005 Da or adjusted depending on MS resolution; and (iv) the choice of the ion with the highest intensity as the representative ion.

For GC/HRMS datasets, we recommend to first filter the dataset to remove unspecific fragments of derivative agents. Then, the same parameters as above can be used, but we recommend choosing the ion with the highest mass among the top highest intensity as representative. As an example, ACorF was also applied to process a GC-MS dataset publicly available on W4M named ‘Algae’ (W4M00004_GCMS-Algae). For GC-MS use, ACorF also allowed for the subdivision of some pc-groups into smaller ones (see supplemental Figure S4). However, the main added value compared to CAMERA is the ability to perform the grouping using annotation (versus 40% annotations in grouped features with CAMERA) and choosing an adequate quantifier ion, more specific than the highest intensity ion.

Different output files are produced for further data analysis steps within workflows. To perform dataset filtration following the ACorF utilisation, we encourage users to work with tools available in the W4M instance. The “Generic Filter” can be used to exclude all lines with a “0” value in the ACorF_filter column of the variableMetadata and to provide the filtered DataMatrix for further statistical analysis in W4M.

Finally, the users can make use of group information for metabolite annotation, especially for LC-MS, by generating sub-files corresponding to the different groups or performing queries in databases using the DataMatrix filtered with representative ions.

## 4. Conclusions

We introduced a new tool, ACorF, which allows identifying and filtering the analytical redundancy within metabolomics LC- and GC-MS datasets. The developed algorithm uses three independent key parameters (any similarity measurement, retention time, and mass difference between features) to group ions part of the same metabolites and the intensity information to propose a representative feature of the group. Finally, as a key element within metabolomics data analysis workflow, this tool is available via the web-based galaxy platform W4M with generic input tabular files and propose different output files for visualisation for further data analysis within workflows.

## Figures and Tables

**Table 1:** Comparison of the CAMERA.annotate and ACorF tool functionalities.

-	CAMERA.annotate (W4M version)	“Analytic Correlation Filtration” (ACorF) Tool
Interface	Galaxy (W4M)	Galaxy (W4M)
Language	R	Perl
Version	Galaxy version 2.1.3	-
Input files	.Rdata output from XCMS Galaxy pre-processing	DataMatrix, variableMetadata and similarity matrix
Parameters	-	-
Mandatory	-	Correlation rate Representative selection method
Optional	Correlation rate RT window determination variables	Mass difference list Retention time tolerance delta
Correlation information	Calculation of correlation is included in the tool	A correlation table has to be obtained before using the tool
Correlation type	Pearson correlation	Any type of correlation are possible
Possibility to set a Correlation threshold	Yes—only for the second step of grouping	Yes
Retention time (RT) window	Calculated for each peak	Defined by a threshold
Parameter settings	Two different parameters ([sigma] and [perfwhm]) are available	The user can set the RT tolerance delta value
Comparison to a mass defect list	Conditioned by obtained group but not used for grouping	When used, directly impact the group determination
Isotope identification	Yes—performed in a previous step	Yes—if the isotope mass difference is included in the list
Existing default list	Yes	Yes
Possibility to upload a personal list	Yes	Yes
Possibility of setting a mass difference tolerance value	No	Yes
Possibility of selecting a representative ion for each group	No	Yes
Output files	variableMetadata with additional columns	variableMetadata with additional columns and a .sif file for network visualisation
Optional output files	EIC for main pc-group visualisation pdf file	-

## Supplementary Materials

The following are available online at https://www.mdpi.com/2218-1989/9/11/250/s1, Figure S1: Total overview of correlation > 0.75 within the Sacurine dataset, Figure S2: Presentation of the 3 input files for the analytic correlation filtration tool. Figure S3. Example of annotation of mass difference between features for early coeluting compounds using ACorF (mass threshold 0.002 Da) in comparison to CAMERA. Figure S4: Bar diagram presenting the number of groups (x-axis) by the group size (y-axis: number of ions per group) obtained from the GC-MS dataset (W4M00004_GCMS-Algae).
