# Supplementary material for: Analytic Correlation Filtration: A New Tool to Reduce Analytical Complexity of Metabolomic Datasets
Source: Metabolites. 2019 Oct 24;9(11):250. doi: 10.3390/metabo9110250 (PMC6918187; doi:10.3390/metabo9110250)

SUPPLEMENTAL DATA:

Supplemental figure 1: Total overview of correlation >0.75 within the Sacurine dataset. Red features are detected as being redundant and tagged as ‘deleted’ by the ACorF tool.

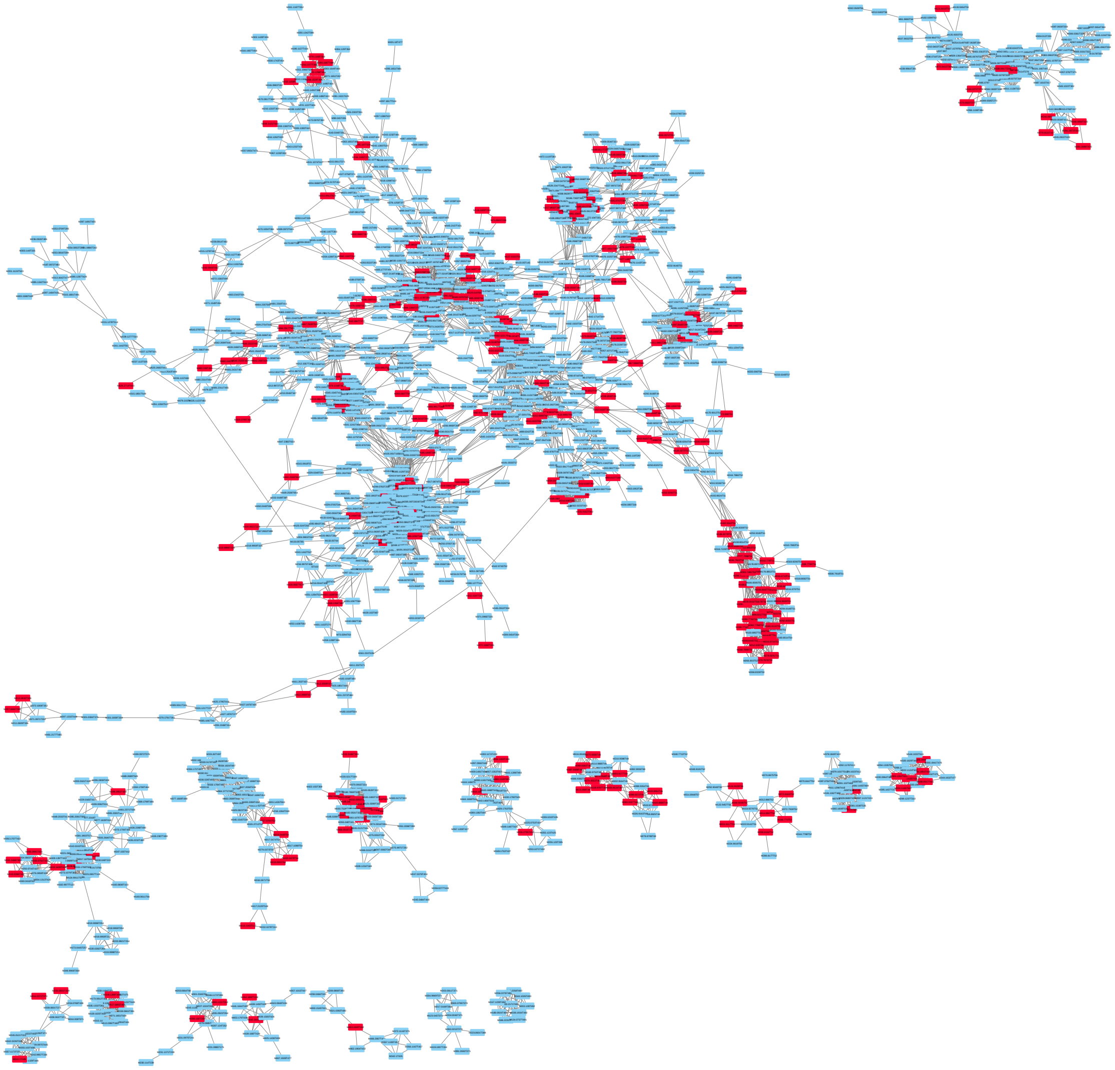

Supplement: Supplementary file 1 [file metabolites-09-00250-s001.zip › Supplemental_1.pdf]
