# Supplementary material for: Analytic Correlation Filtration: A New Tool to Reduce Analytical Complexity of Metabolomic Datasets
Source: Metabolites. 2019 Oct 24;9(11):250. doi: 10.3390/metabo9110250 (PMC6918187; doi:10.3390/metabo9110250)
Supplement: Supplementary file 1 [file metabolites-09-00250-s001.zip › Supplemental_2.pdf]

Supplemental figure 2: Presentation of the 3 input files for the analytic correlation filtration tool.

dataMatrix	HU_neg_011_b2	HU_neg_014_b2	HU_neg_015_b2	HU_neg_017	HU_neg_018	HU_neg_019_b2	HU_neg_020
M57.9755T69	83357.47884	65755.66285	1715221.391	269202.6962	291351.1943	627193.05	345432.8919
M59.0137T249	14413.54965	9092.148822	67653.3694	12188.25765	198392.0939	24067.43472	0
M59.0137T58	10932.64749	8848.391489	23868.80878	0	96970.38763	12730.73992	0
M61.9882T56	19413716.39	14005779.79	7653078.016	6024721.158	17992838.8	9781661.578	6052777.822
M70.9459T55	21744.96781	81920.64907	133903.64	76864.40655	38973.62343	44041.17485	135466.0025
M71.0137T88	2327.376897	2859.696349	70684.91186	13135.62237	13738.05842	4471.057661	8445.549791
M71.0501T244	9427.686141	6551.573193	69425.55564	9839.619588	117969.2533	26690.57273	30478.53685
M72.9428T55	15447.27241	44528.09221	64764.39928	51074.46653	32631.58396	29021.33022	93043.19587
M73.0293T150	483891.7412	231477.1943	563917.3413	239274.8959	1859432.222	1022119.901	409300.8155
M73.0294T63	16269.94995	9451.470584	10745.00609	13636.73721	27670.64808	33114.41593	12758.00886
M74.0244T316	0	13447.80481	31575.92519	9591.994048	14374.89211	16895.37739	19053.43023
M75.0086T59	727684.4725	565400.0259	1337109.604	224231.3576	2326480.193	1270932.728	519624.6958
M77.0063T60	36622.90886	10537.09347	36649.69969	45987.87768	69730.50937	93832.50288	23332.46162
M78.9186T54	20919.10588	6390.10913	30389.96924	33555.10705	0	0	40909.12752
M78.9589T54	242311.453	285486.315	75771.69495	97057.26958	265730.5902	239873.168	30693.43104
M78.9856T59	157576.8551	125182.5429	52415.99127	42115.76713	114159.0776	141379.6895	30553.63146
M79.9572T381	738044.9704	79277.10423	175118.3555	27655.76942	622686.3401	467886.2744	228563.7189
M80.9747T54	4106852.353	6117132.465	9674659.28	7020611.902	5450793.239	5698352.2	8436434.983
M82.9717T54	1969268.659	2980500.789	4875748.778	3685446.271	2536322.95	2835334.064	4363609.969

A: Data matrix containing intensities of each variable per sample

variableMetadata	mz	mzmin	mzmax	rt	rtmin	rtmax	npeaks
M57.9755T69	57.97545016	57.97513446	57.97578378	69.0312573	67.5911153	70.74979638	167
M59.0137T249	59.01373455	59.01296665	59.01407715	248.7138883	246.1729223	249.7438125	94
M59.0137T58	59.01369322	59.01330927	59.01406964	58.07568876	56.48748707	62.5180627	24
M61.9882T56	61.98818999	61.98782114	61.98874327	56.14519904	54.37507075	76.62749333	226
M70.9459T55	70.94587667	70.94554589	70.94622332	54.60254236	52.81379267	59.62247161	189
M71.0137T88	71.01366844	71.01321617	71.01401565	87.58651643	84.60303232	89.2023364	25
M71.0501T244	71.0500901	71.04941118	71.05031641	243.8300073	241.0351217	247.4337427	69
M72.9428T55	72.94281507	72.94281507	72.94317809	54.56092075	52.93922437	59.62247161	159
M73.0293T150	73.0292879	73.02881625	73.02988747	149.6937378	143.6401655	151.7745755	157
M73.0294T63	73.0294158	73.02881625	73.02988747	62.52738316	61.37867656	74.19694229	76
M74.0244T316	74.02440658	74.02392525	74.02512859	316.2099478	314.8735248	318.1114984	97
M75.0086T59	75.00857277	75.00759084	75.00930922	59.4166567	53.96198255	98.33325008	257
M77.0063T60	77.00631614	77.00620256	77.00671327	60.256716	58.82394188	61.17047686	149
M78.9186T54	78.9185754	78.91778063	78.91898898	53.67792136	52.7383119	55.09067865	69
M78.9589T54	78.9588594	78.95806403	78.95953846	53.61965667	48.83801792	60.91688615	248
M78.9856T59	78.98564412	78.98537886	78.98605828	59.20101874	58.62008304	60.55603521	35
M79.9572T381	79.9572298	79.95634308	79.95742376	380.6272464	375.0510089	390.9198887	194
M80.9747T54	80.97465687	80.97410618	80.97575826	54.19622623	51.71408211	67.51222163	234
M82.9717T54	82.97170627	82.97142068	82.9718696	54.2626734	52.21379721	55.92278124	225
M85.0292T64	85.02920419	85.02920418	85.0293751	64.19271037	63.09620814	64.66357392	210

B: Variable metadata containing descriptive additional metadata of variables (*e.g.* m/z, retention time).

name	M57.9755T69	M59.0137T249	M59.0137T58	M61.9882T56	M70.9459T55	M71.0137T88	M71.0501T244
M57.9755T69	1	0.069706364	0.068036382	0.09630657	-0.041468696	0.16961881	0.161209567
M59.0137T249	0.069706364	1	0.197061108	0.031477456	-0.008549106	-0.014784634	0.474054439
M59.0137T58	0.068036382	0.197061108	1	-0.035144676	0.029241133	0.406008205	0.271500336
M61.9882T56	0.09630657	0.031477456	-0.035144676	1	-0.091578289	-0.06477617	0.005295074
M70.9459T55	-0.041468696	-0.008549106	0.029241133	-0.091578289	1	-0.03650672	-0.047764902
M71.0137T88	0.16961881	-0.014784634	0.406008205	-0.06477617	-0.03650672	1	-0.047462231
M71.0501T244	0.161209567	0.474054439	0.271500336	0.005295074	-0.047764902	-0.047462231	1
M72.9428T55	-0.03255863	-0.019732269	0.017280549	-0.086204146	0.993035514	-0.039992935	-0.061072947
M73.0293T150	0.167204678	0.385535367	0.384689114	0.126156615	-0.044935808	0.122782003	0.44650281
M73.0294T63	0.20701375	0.086785463	0.341982295	0.083411774	-0.030771063	0.155792041	0.201345311
M74.0244T316	0.025253218	0.11505731	-0.002460977	0.051770257	0.004411937	0.024891512	0.1599277
M75.0086T59	0.459145487	0.190896229	0.293863843	0.290921153	0.000376437	0.099330952	0.233858684
M77.0063T60	0.73117753	0.00771157	0.065812289	0.132338201	-0.013703533	0.091391889	0.043369203
M78.9186T54	0.093616588	-0.060358118	-0.104374479	-0.062826433	0.10298002	-0.101634027	-0.100836923
M78.9589T54	0.136860764	-0.023849582	-0.066576616	0.279060794	-0.157412063	-0.069205675	0.058811834
M78.9856T59	0.151273591	0.054201921	0.118239785	0.262194947	-0.163746403	-0.095391046	0.058958032
M79.9572T381	0.014794935	0.12459925	0.194416555	0.166065373	0.026558816	-0.052700374	0.354291416
M80.9747T54	0.534936369	-0.045594376	-0.001455596	0.164747378	0.065822071	0.035702963	-0.091250877
M82.9717T54	0.51803599	-0.050989878	-0.005972614	0.13884301	0.08761496	0.029917699	-0.106838672
M85.0292T64	0.19192721	0.301326774	0.486718522	0.207831107	-0.006192991	0.207884217	0.481588338
M85.0292T110	0.086578163	0.222510766	0.319608996	0.171898332	-0.104909565	0.146972465	0.34265265

C: Similarity matrix representing pair-wise similarity within the dataset
