# Supplementary material for: Analytic Correlation Filtration: A New Tool to Reduce Analytical Complexity of Metabolomic Datasets
Source: Metabolites. 2019 Oct 24;9(11):250. doi: 10.3390/metabo9110250 (PMC6918187; doi:10.3390/metabo9110250)

Supplemental figure 3: Example of annotation of mass difference between features for early co-eluting compounds using ACorF (mass threshold 0.002 Da) in comparison to CAMERA

ions	pc-group	ACorF_groups	Identification
p203.052T0.9	10	group23	glucose / mannose / fructose
p97.0284T0.9	10	group23	
p441.074T0.9	10	group23	
p145.049T0.9	10	group23	
p383.116T0.9	10	group23	
p325.113T0.9	10	group23	
p109.028T0.9	10	group23	
p69.0335T0.9	10	group23	
p148.060T0.9	10	group311	glutamic acid
p231.145T0.9	10	group319	glutamyl-threonine
p227.125T0.9	10	group320	unknown

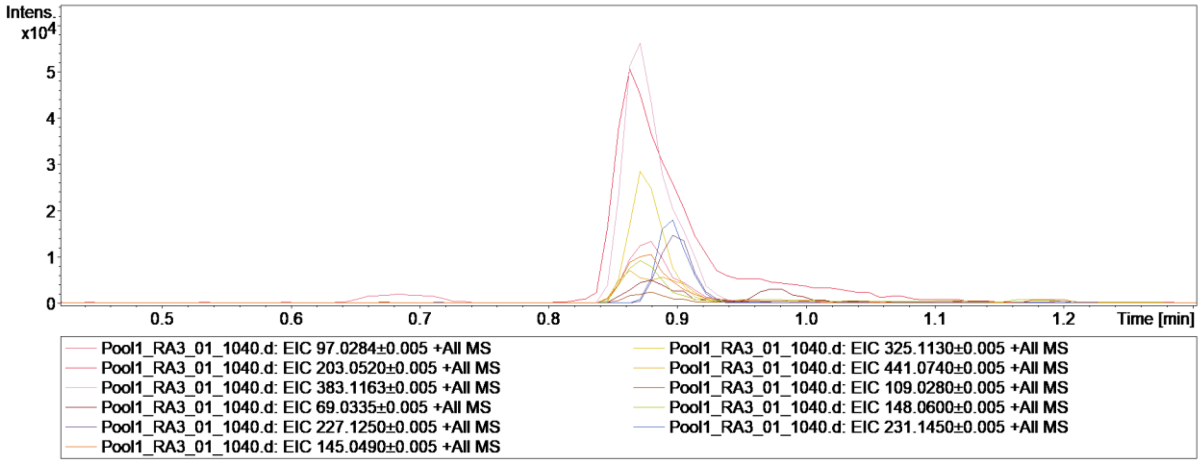

Supplement: Supplementary file 1 [file metabolites-09-00250-s001.zip › Supplemental_3.pdf]
