# Supplementary material for: Analytic Correlation Filtration: A New Tool to Reduce Analytical Complexity of Metabolomic Datasets
Source: Metabolites. 2019 Oct 24;9(11):250. doi: 10.3390/metabo9110250 (PMC6918187; doi:10.3390/metabo9110250)

Supplemental figure 4: Bar diagram presenting the number of groups (x-axis) by the group size (y-axis: number of ions per group) obtained from the GC-MS dataset (W4M00004_GCMS-Algae).

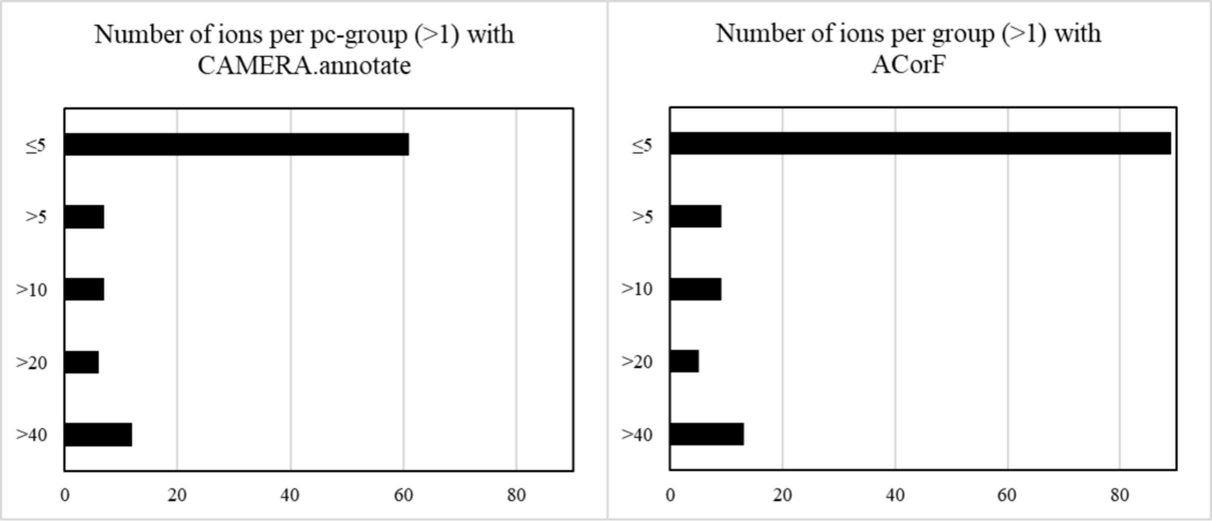

Supplement: Supplementary file 1 [file metabolites-09-00250-s001.zip › Supplemental_4.pdf]
